# Novel Rearrangements in the Reactions Directed Toward Preparation of Spiro-*N*,*N*-ketals: Reactions of Naphthalene-1,8-diamine with Ninhydrin and Isatin

**DOI:** 10.3390/molecules171213879

**Published:** 2012-11-22

**Authors:** Motoko Akita, Hideyuki Seto, Reiko Aoyama, Junko Kimura, Keiji Kobayashi

**Affiliations:** Graduate School of Science, Josai University, Sakado, Saitama 350-0295, Japan

**Keywords:** spiro-*N*,*N*-ketals, perimidine, diazapleiadiene, prototropic tautomerism, X-ray structure determination

## Abstract

Spiro-*N,N*-ketal **5**, consisting of a phthaloperine heterocyclic ring and a naphtha[1,8-ef][1,4]diazepine ring, was obtained along with spiro-*N,N*-ketal **2** via 2,2-condensation in the reaction of ninhydrin with naphthalene-1,8-diamine. Their molecular structures were elucidated by X-ray crystal structural analysis. Aside from these spiro compounds, the diazapleiadiene compound **3** formed by 1,2-condensation and the 1,4-isoquinolinedione compound **4** arising from ring expansion were isolated. When isatin was reacted with naphthalene-1,8-diamine, spiro-*N*,*N*-ketal **6** and the two 1*H*-perimidine-based compounds **7** and **8** were isolated. Compound **8** was revealed to undergo a fast dynamic prototropic tautomerization in solution. Plausible mechanisms of the formation of the products are proposed.

## 1. Introduction

Among modifications of spiroketals, the aza-aza spiro systems, designated as spiro-*N,N*-ketals or spiroaminals [1], have received much less attention than their oxygenated analogues. The spiro-*N,N*-ketals obtained by the reaction of ninhydrin and diamines, e.g., 1,3-diphenylspiro[imidazolidine-2,2'-indan]-1',3'-dione (**1**) [2,3,4], exhibit characteristic deep-red colorations [2,3]. Despite the apparent absence of extended π-conjugation, the occurrence of such long-wavelength absorption is attributed to spiro conjugation [5,6] through intramolecular charge-transfer interactions between the 1,3-indandione moiety as the acceptor unit and aromatic diamine as the donor unit [7]. With this background in mind, we prepared spiro-*N,N*-ketals by the reactions of ninhydrin (2,2'-dihydroxy-1*H*-indene-1,3(**2**H)-dione) and isatin (1*H*-indole-2,3-dione) with diamines. When naphthalene-1,8-diamine was used as the diamine, unexpected rearranged products were obtained, along with new spiro-*N,N*-ketals. Herein, we report the outcome of these reactions and the characteristic structural and spectroscopic properties of the products. 

## 2. Results and Discussion

### 2.1. Reaction with Ninhydrin

**Scheme 1:**
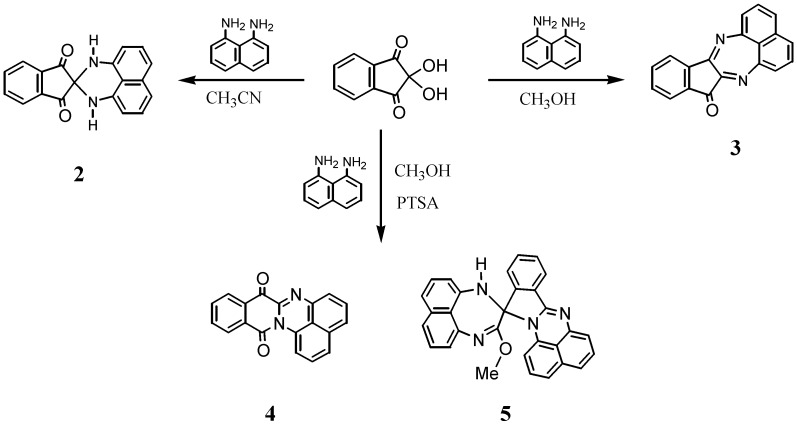
Reaction of ninhydrin with naphthalene-1,8-diamine.

**Figure 1:**
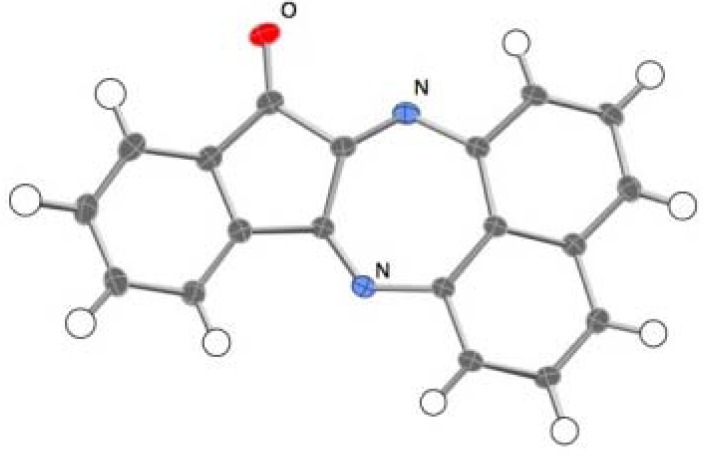
ORTEP representation of **3**.

The reactions of ninhydrin with primary diamines generally afford 1,2-condensed compounds based on the indenopyrazine framework [8,9,10]. Spiro-*N,N*-ketals have been prepared using a secondary diamine, *N*,*N'*-dimethyl-1,8-naphthalenediamine [7], while no analogous reactions using primary diamines have yet been described. Thus, when 1,8-naphthalenediamine was allowed to react with ninhydrin in acetonitrile for 24 h, spiro-*N,N* acetal **2** was isolated in 18% yield as red crystals. No products other than **2** could be isolated, because the resulting solids consist of a complex mixture that entrapped upon column chromatography. The structure of **2** was readily identified by ^1^H-NMR spectroscopy, where it displays only one set of doublet-triplet-doublet signal patterns, indicating a symmetric structural environment of the naphthalene ring. In accordance with the ^1^H-NMR spectrum, the ^13^C-NMR spectrum exhibits eleven signals, including a signal at 197.0 ppm assignable to a carbonyl carbon. Furthermore, the structure of **2** was unambiguously determined by X-ray structural analysis, as will be described later in other sections. In contrast to the reaction in acetonitrile, the reaction in methanol led to the isolation of a 1,2-condensed product, the diazapleiadiene derivative **3**, in 42% yield (Scheme 1). The structure of **3** was elucidated on the basis of NMR spectroscopy and mass spectral analysis and eventually confirmed by X ray crystallographic analysis (Figure 1). 

Next, the reaction in methanol was conducted by adding a small amount of *p*-toluenesulfonic acid, which resulted in the formation of the unexpected rearranged products **4** and **5** in 23% and 38% yields, respectively. The structures of these compounds were elucidated by spectroscopic data; that of **5** was additionally confirmed by X-ray crystallographic analysis. The ^1^H-NMR spectrum of **4** shows two sets of doublet-triplet-doublet signal patterns assignable to the naphthalene ring and doublet-triplet-triplet-doublet signals due to the benzene ring of the isoquinoline framework. One of the doublet signals occurs at a low field at 8.6 ppm, indicating its location to be spatially close to the carbonyl group. The ^13^C-NMR spectrum shows two signals at 174.9 and 160.0 ppm, assignable to carbonyl carbons, among which the latter is ascribed to an amide carbon. The mass spectrum shows abundant *m/z* = M+2 ions typical of many quinones [11]. From this spectral information, the structure of **4** could be deduced. 

**Figure 2:**
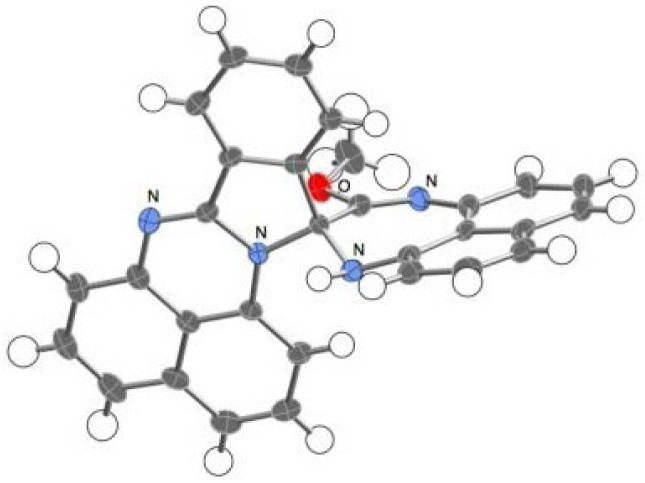
ORTEP representation of **5**. Enclathrated acetonitrile molecules are omitted for clarity.

The novel spiro compound **5**, an orange crystalline solid, has a molecular weight of 452 in the mass spectrum and displays four sets of doublet-triplet-doublet signal patterns and one set of doublet-triplet-triplet-doublet patterns in its ^1^H-NMR spectrum. A signal assignable to a methoxy substituent is observed at 3.68 ppm. Eventually, the structure of **5** was confirmed by X-ray analysis to be a spiro-conjoined compound, as noted in a later section (Figure 2). 

**Scheme 2:**
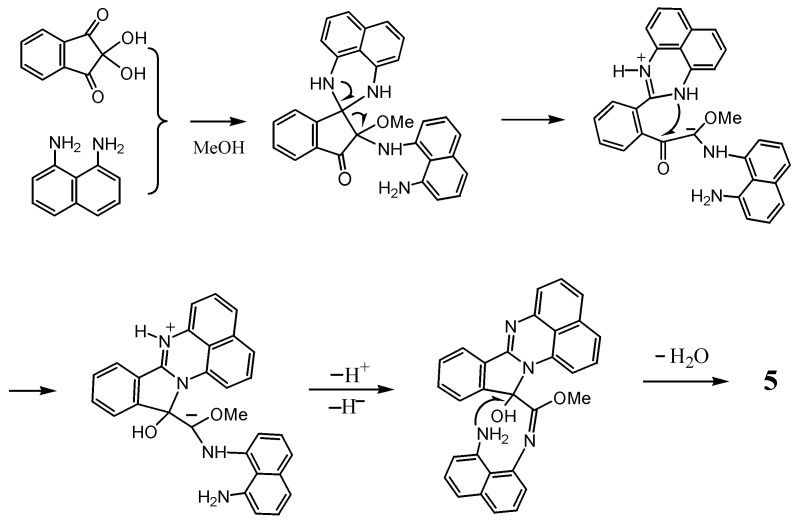
Plausible mechanism of formation of **5**.

The formation of **4** and **5** entails rearrangements. The formation of **4** could be interpreted by C-N bond migration from the spiro carbon to the carbonyl carbon of **2** as the precursor. For **5**, its possible formation mechanism is shown in Scheme 2. An acid catalyst activates the electrophilic reactivity, not only at C2 position, but also at the less reactive C1, allowing the latter to react with diamine and methanol. The intermediate thus formed would proceed to ring opening retaining a 1*H*-perimidine framework and the subsequent ring closure with the intervention of another diaminonaphthalene ring. This mechanism is analogous to that proposed for the reaction of 2-acyl-1,3-indandione with naphthalene-1,8-diamine to give 10-methyl-10-naphthaloperinol [12].

It should be noted that the formation of both **4** and **5** is formally a dehydrogenation reaction, judging from the stoichiometry of such formation. Although we could not identify the oxidation agent required in this reaction, the most plausible one is considered to be ninhydrin, based on the result that **4** was obtained even when the reaction was carried out under nitrogen. Ninhydrin itself could participate in oxidation [13], because the C2 position of ninhydrin has “umpolung” ability. The carbocation at C2, resulting from the release of the OH group as an anion, should be stabilized by accepting electrons to be converted to a carbanion.

### 2.2. Reactions with Isatin

**Scheme 3:**
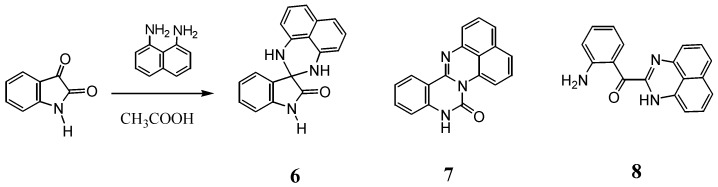
Reaction of isatine with naphthalene-18-diamine.

The reaction of isatin with a few primary diamines has been reported to give 3,3-spiro and 2,3-condensed compounds [14,15,16,17,18,19]. The reaction of isatin with naphthalene-1,8-diamine has also been described in the literature [20,21,22,23]. Here, we reinvestigated this reaction under the conditions involving the use of acetic acid as the solvent in the presence of a catalytic amount of *p*-toluenesulfonic acid. The three products **6**–**8** were isolated in 45%, 9% and 6% yields, respectively (Scheme 3). These structures were revealed from their NMR, IR, and mass spectra and confirmed for **7** and **8** by X-ray crystallographic analyses. 

**Figure 3:**
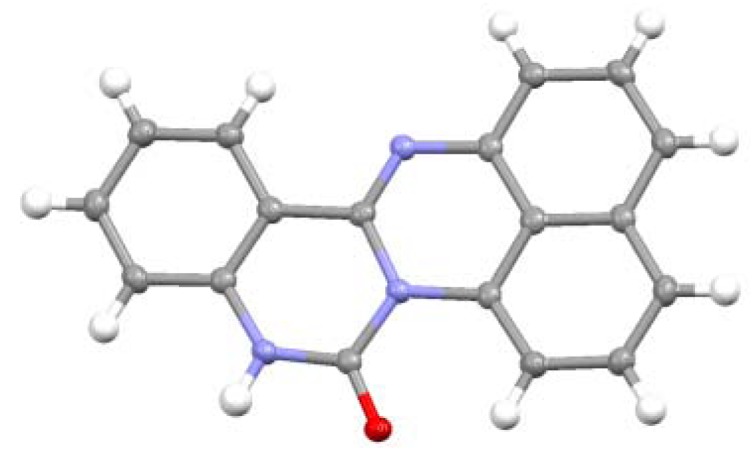
ORTEP representation of **7**.

The ^1^H and ^13^C-NMR spectra of spiro-*N,N*-ketal **6** showed good agreement with those reported for 1',3'-dispiro[indoline-3,2'-perimidin]-2-one [22]. Compound **7** was identified as quinazolino[3,4-a]perimidin-6(5*H*)-one on the basis of X-ray analysis results (Figure 3), and its spectral data were identical to those reported in the literature [24]. 

**Figure 4:**
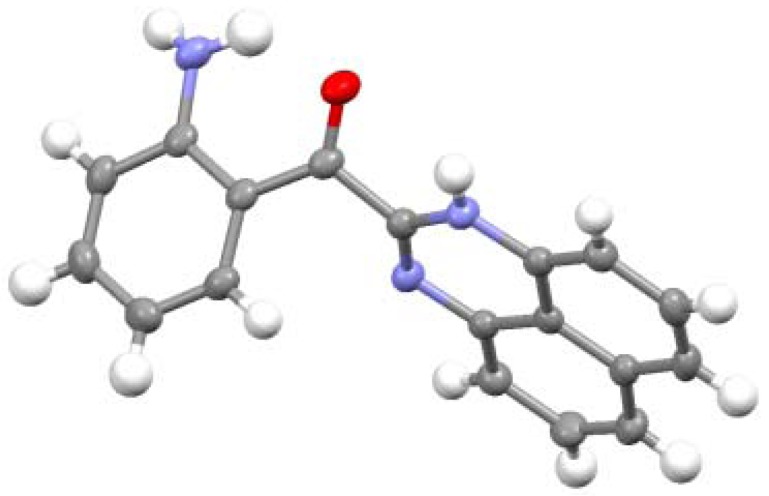
ORTEP representation of **8**.

**Figure 5:**
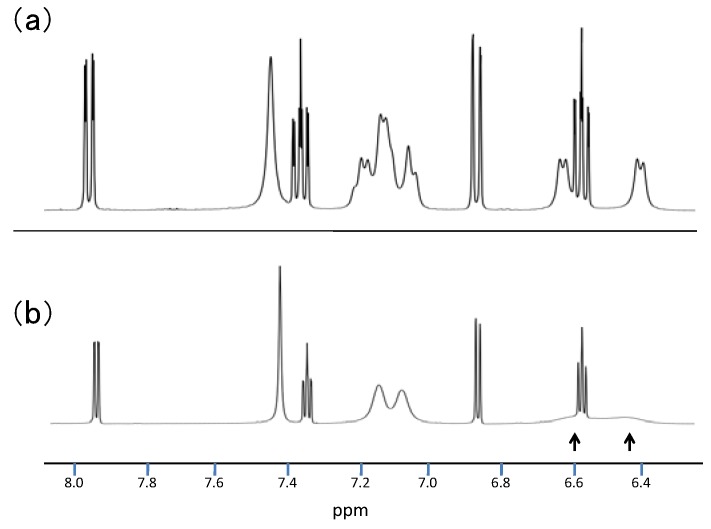
(**a**) ^1^H-NMR spectrum of **8** in DMSO-d_6_. (**b**) ^1^H-NMR spectrum of **8** after addition of trace amount of D_2_O in DMSO-D_6_.

**Scheme 4:**
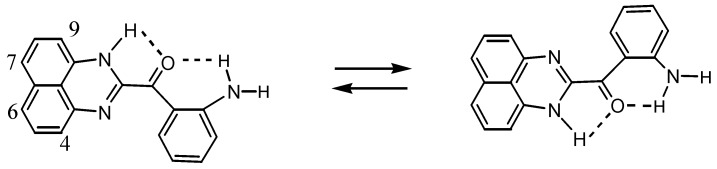
Prototropic dynamic tautomerization of **8**.

The structure of **8** was determined by X-ray analysis (Figure 4). The carbonyl oxygen forms an intramolecular hydrogen bond with the NH proton of the perimedine moiety (O---N: 2.784 A) and at the same time with the amino NH protons (O---N: 2.783 A), that is, it forms a bifurcated hydrogen bond. The ^1^H-NMR spectrum of **8** in DMSO-D_6_ exhibits proton signals of the amino-substituted benzene ring as sharp distinct signals in a doublet-triplet-triplet-doublet pattern, while those of the perimidine ring occur as markedly broad signals (Figure 5a). This observation indicates the involvement of dynamic prototropic tautomerization in the perimidine ring, as depicted in Scheme 4. The averaging of the signals of the C4 and C9 protons at approximately 6.4 and 6.6 ppm is enhanced by adding a trace amount of deuterated water (D_2_O), indicating that the observed prototropy is an intermolecular phenomenon (Figure 5b). The NH proton of the perimidine ring and one of the NH_2_ protons of the aniline ring are not observed, probably due to rapid exchange, whereas one of the amino protons appears at 7.43 ppm as a relatively sharp signal. This observation suggests that only the latter proton participates in the intermolecular hydrogen bonding with DMSO. A similar dynamic prototropic behavior accompanied by the carbon–carbon bond rotation has been found for an analogous perimidine system including the intramolecular NH----O=C hydrogen bonding system [25] and for a pyridine compound including the NH---N hydrogen bonding system [26].

**Scheme 5:**
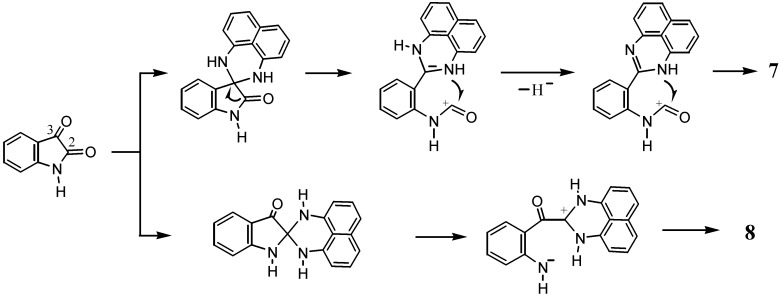
Possible mechanism of the formation of **7** and **8**.

Taking into consideration that both **7** and **8** possess the 1*H*-perimidine framework, the spiro-conjoining of 1,8-naphthalenediamine to the C3 carbon of isatin would be responsible for the formation of **7**, while the spiro-conjoining at the C2 carbon would result in the formation of **8** (Scheme 5). The electrophilic reactivity of the C3 carbonyl group in isatin is higher than that of the C2 carbon. In the presence of an acid catalyst, the less reactive C2 position could be activated to undergo reactions. It should be noted that the reaction leading to **7** again entails dehydrogenation. Probably, atmospheric oxygen participates in the formation of **7**. 

### 2.3.Structural Characteristics of the New Spiro-N,N-ketals

**Figure 6:**
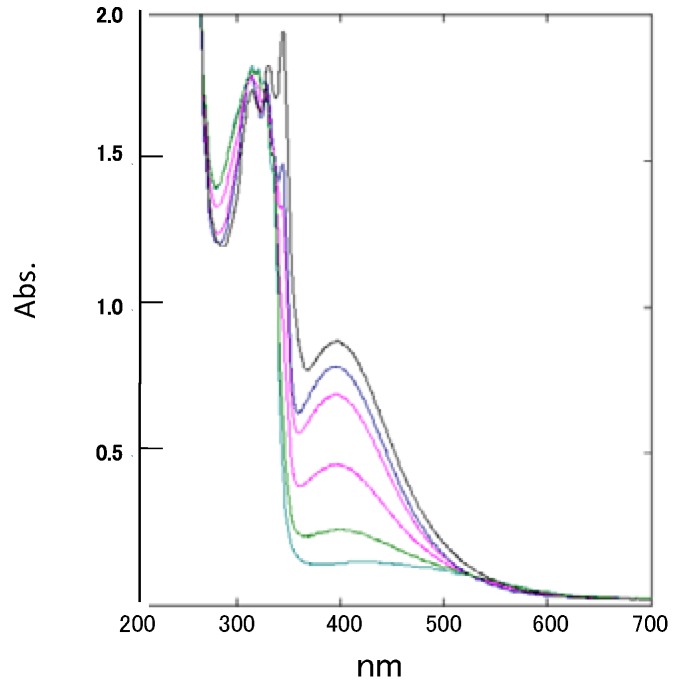
UV/Vis spectra of **2**, showing the decrease in intensity of its 396 nm band upon addition of hydrochloric acid (in methanol).

The UV/Vis spectrum of spiro-*N,N*-ketal **2**, which is red in the solid state, exhibited an absorption maximum at 396 nm (ε = 440) with end-absorption bands up to 600 nm. No such bands are observed for the two component subchromophores, indicating that the absorption maximum at 396 nm results from spiro-conjugation between two chromophores. This absorption band corresponds well to that observed for the *N,N*-dimethyl derivative of **2** reported by Maslak [7]. The intensity of the 396 nm band gradually decreased with the addition of dilute hydrochloric acid (Figure 6), suggesting that this absorption band is attributed to a n-π* transition and intramolecular charge-transfer interaction exerted through unpaired electrons on nitrogen atoms. 

**Figure 7:**
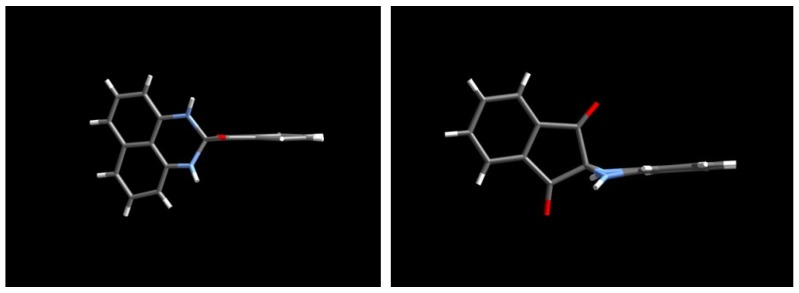
Side views of molecular structure of **2**.

The X-ray analysis result of **2** is not sufficient to provide n accurate structural parameters because of the poor crystallinity of the single crystal employed for data collection; however, it is satisfactory for revealing the gross structure of **2**. The molecules of **2** are packed in the *P*-1 space group and there are two independent molecules, A and B, in a unit cell. In these two molecules, the 1,3-indandione moiety is planar, while the 2,3-dihydro-1*H*-perimidine ring is bent along the line connecting the two nitrogen atoms. Thus, the nitrogen atoms are slightly pyramidalized. The plane made by the two nitrogen and spiro-carbon atoms constitutes an approximately perpendicular geometry with respect to the plane made by the two carbonyl carbon and spiro-carbon atoms (Figure 7). 

The spiro compound **5** exhibited an orange color reminiscent of spiro-conjugation, showing absorption maxima at λ_max_ = 459 (ε = 2041), 434 (ε = 2500), and 406 (ε = 2340) nm. Single crystals of **5** suited for X-ray studies were obtained by a slow evaporation of the solvent from MeCN solution. An X-ray crystallographic analysis revealed that the crystals are solvated by MeCN molecules in a ratio of **5**:MeCN = 2:1. These solvent molecules are embedded with disorder in a tunnel-like column along the *b*-axis. One of the spiro components, which includes a diazepine ring, is planar, while the other component, consisting of a naphthoperimidine moiety, is slightly bent. The geometry of the spiro-carbon is close to the tetrahedral structure. 

## 3. Experimental

### General

All the melting points were determined using a Yanaco MS-500V apparatus and are uncorrected. The ^1^H-NMR (400 MHz) and ^13^C-NMR (100.5 MHz) spectra were recorded using an Agilent-400MR spectrometer. Chemical shifts are given in δ values (ppm) using TMS as the internal standard. Coupling constants (*J*) are Hz. FTIR spectra were measured with a Shimadzu 8200PC spectrophotometer. Mass spectra were taken on a Shimadzu GCMS-QP5050A mass spectrometer. Elementary combustion analysis was performed using a Yanaco CHN CORDER MT-6 analyzer. All reactions were monitored by TLC employing 0.25 mm silica gel plates (Merck 60F 254). 

### 3.1. Reaction with Ninhydrin

#### 3.1.1. In Acetonitrile

A solution of ninhydrin (1.11 g, 6.23 mmol) and naphthalene-1,8-diamine (0.98 g, 6.23 mmol) in acetonitrile (100 mL) was stirred at room temperature for 24 h. Aqueous work up, extraction with chloroform, and drying over anhydrous MgSO_4_ afforded a reddish solution. After the solvent was removed under reduced pressure, the residual solid was chromatographed on silica gel with dichloromethane as the eluent to give *1',3'-Dihydrospiro[indene-2,2'-perimedine]-1,3-dione* (**2**, 0.33 g, 18%); mp 186–188 °C; ^1^H-NMR (CDCl_3_): *δ* 8.00 (4H, m of A_2_B_2_), 7.35 (4H, t, *J *= 8.3), 7.31(2H, d, *J *= 7.2), 6.71 (4H, d, *J* = 7.2), 4.60 (2H, br-s); ^13^C-NMR (DMSO-d_6_): *δ *66.2, 104.0, 109.6, 115.1, 122.4, 125.7, 132.3, 135.8, 138.1, 138.6, 197.0; MS: *m/z* 300 (M^+^); Anal. Calcd for C_19_H_12_O_2_N_2_: C 75.98, H 4.03, N 9.33%. Found: C 76.21, H 3.87, N 9.53%. 

#### 3.1.2. In Methanol

To a solution of ninhydrin (1.13 g, 6.35 mmol) in methanol (50 mL) was added naphthlene-1,8-diamine (0.98 g, 6.23 mmol) dissolved in methanol (50 mL). The mixture was stirred for 24 hr at room temperature, in which brown solids separated out. The solids were filtered off and washed with methanol, acetonitrile, and benzene, successively. The resulting solids (crude yield ca. 51%) were recrystallized from acetonitrile to give *5H-indeno[1,2-b]naphtho[1,8-ef][1,4]diazepin-5-one* (**3**, 0.74 g, 42%); mp 240–244 °C; ^1^H-NMR (CDCl_3_): *δ* 8.11 (1H, d, *J *= 7.6), 8.02 (1H, d, *J *= 7.6), 7.84 (1H, t, *J *= 7.4), 7.74 (1H, t, *J *= 7.6), 7.60 (1H, d, *J *= 7.3), 7.52 (1H, d, *J *= 8.2), 7.42 (1H, d, *J *= 8.1), 7.39 (1H, d, *J *= 7.3), 7.24 (2H, d, *J *= 7.8); ^13^C-NMR (DMSO-d_6_): *δ* 123.0, 123.6, 127.9, 128.1, 128.3, 129.1, 130.7, 132.3, 133.4, 133.5, 134.9, 136.8, 138.0, 138.9, 143.1, 143.6, 143.9, 186.7; IR (KBr) 1717 cm^−1^; MS *m/z* 282 (M^+^); Anal. Calcd for C_19_H_10_ON_2_: C 80.83, H 3.58, N 9.92%. Found: C 80.65, H 3.32, N 9.70%.

#### 3.1.3. In Methanol with *p*-Toluenesulfonic Acid

To a solution of ninhydrin (1.13 g, 6.35 mmol) and *p*-toluenesulfonic acid (0.01 g) in methanol (50 mL) was added naphthlene-1,8-diamine (1.00 g, 6.35 mmol) dissolved in methanol (50 mL). The mixture was stirred for 24 hr at room temperature. Aqueous work up, extraction with chloroform, and drying over anhydrous MgSO_4_ afforded a dark solution. After the solvent was removed under reduced pressure, the residual solid was chromatographed on a silica gel with benzene as the eluent to give 8H,13H-isoquinolino[2,3-a]perimidine-8,13-dione (**4**, 0.43 g, 23%) and 3'-methoxy-3a1,6a-dihydro-1'H-spiro[isoindolo[2,1-a]perimidine-12,2'-naphtho[1,8-ef][1,4]diazepine] (**5**, 0.54 g, 38%).

*Compound* **4**: mp 172–175 °C; ^1^H-NMR (CD_3_CN): *δ* 8.60 (1H, d, *J *= 7.9), 8.43 (1H, d, *J *= 7.0), 8.23 (1H, d, *J *= 8.2), 7.99 (1H, t, *J *= 7.6), 7.91 (1H, t, *J *= 7.6), 7.67 (1H, d, *J *= 8.2), 7.63 (1H, d, *J *= 8.2), 7.51 (1H, t, *J *= 7.9), 7.49 (1H, t, *J *= 8.2). 7.39 (1H, d, *J *= 7.3); ^13^C-NMR (DMSO-d_6_): *δ* 115.5, 121.6, 122.5, 123.8, 126.5, 126.7, 128.2, 128.5, 129.3, 131.3, 131.6, 133.3, 134.0, 134.4, 135.7, 138.0, 143.8, 160.6, 174.9; IR (KBr): 1717, 1684 cm^−1^; MS *m/z* 298 (M^+^); Anal. Calcd for C_19_H_10_O_2_N_2_: C 76.49, H 3.38, N 9.39%. Found: C 76.86, H 3.01, N 9.57%.

*Compound* **5**: mp 264 °C; ^1^H-NMR (CD_3_CN): *δ* 7.93 (1H, d, *J *= 7.6), 7.82 (1H, d, *J *= 8.2), 7.68 (1H, d, *J *= 6.1), 7.58 (1H, t, *J *= 7.3), 7.53 (1H, t, *J *= 7.4), 7.25 (1H, m), 6.97 (1H, d, *J *= 6.1), 6.72 (1H, d, *J *= 7.0), 6.62 (2H, d, *J *= 7.6); ^13^C-NMR (CDCl_3_): *δ* 54.1, 81.1, 104.1, 115.9, 116.2, 119.7, 120.4, 121.3, 121.4, 121.5, 122.5, 122.6, 126.6, 126.8, 127.1, 127.7, 128.3, 128.4, 128.5, 129.9, 131.3, 131.4, 135.0, 135.2, 136.4, 138.0, 140.5, 142.8, 143.9. IR (KBr): 3420, 1379, 754 cm^−1^; MS: *m/z* 452 (M^+^); Anal. (solids desolvated *in vacuo*) Calcd for C_30_H_20_ON_4_: C 79.62, H 4.46, N 12.38. Found: C 79.23, H 4.41, N 12.58.

### 3.2. Reaction with Isatin

To a solution of isatin (1.00 g, 6.80 mmol) in acetic acid (50 mL) was added naphthalene-1,8-diamine (1.00 g, 6.33 mmol) and *p*-toluenesulfonic acid (0.01 g) dissolved in acetic acid (60 mL). The mixture was refluxed for 3 h with stirring. The resulting mixture was poured into water, extracted with chloroform, washed with water four times, and dried over anhydrous MgSO_4_. After solvent was removed under reduced pressure, the residual solid was chromatographed on a silica gel with chloroform as the eluent to give 1',3'-dihydrospiro[indoline-3,2’-perimidin]-2-one (**6**, 0.82 g, 45%), quinazolino[3,4-a]perimidin-6(5H)-one (**7**, 0.16 g, 9%), and *2-(2-aminobenzoyl)-1*H*-perimidine* (**8**, 0.11 g, 6%). Compounds **7** and **8** were recrystallized from ethyl acetate and MeCN, respectively, to yield single crystals suited for X-ray crystal structure analysis.

*Compound*** 6**: mp 203–206 °C [27]; ^1^H-NMR (DMSO-d_6_): *δ* 10.28 (1H, s), 7.34 (1H, d, *J *= 7.0), 7.33 (1H, t, *J *= 7.8), 7.14 (2H, t, *J *= 7.7), 7.08 (2H, s), 7.03 (1H, t, *J *= 7.1), 6.97 (2H, d, *J *= 7.0), 6.87 (1H, d, *J *= 7.6), 6.45 (2H, d, *J *= 8.0) ppm. ^13^C-NMR (DMSO-d_6_): *δ *67.4, 104.9, 109.6, 111.2, 115.5, 121.7, 125.2, 126.6, 129.6, 130.4, 133.4, 140.4, 142.5, 177.3. IR (KBr): 1716, 1601 cm^−1^; MS m/z 287 (M^+^); Anal. Calcd for C_18_H_13_ON_3_: C 75.24, H 4.56, N 14.63. Found: C 74.97, H 4.34, N 14.29. 

*Compound* **7**: mp 292–294 °C (lit. 291–293 °C [23]); ^1^H-NMR (DMSO-d_6_): *δ* 11.25 (1H, s), 8.20 (1H, d, *J *= 7.2), 8.13 (1H, d, *J *= 6.7), 7.55 (1H, t, *J *= 6.8), 7.48 (1H, d, *J *= 7.4), 7.37 (2H, t, *J *= 6.1), 7.35 (1H, d, *J *= 8.0), 7.17 (1H, t, *J *= 7.1), 7.07 (1H, d, *J* = 8.0), 6.98 (1H, d, *J *= 6.8); ^13^C-NMR (DMSO-d_6_): 95.3, 113.9, 114.4, 115.2, 116.9, 121.9, 122.2, 122.6, 127.0, 127.8, 133.3, 133.9, 137.5, 139.3, 146.9, 148.2, 174.8. IR(KBr): 1699 cm^−1^; MS *m/z* 285 (M^+^); Anal. Calcd for C_18_H_11_N_3_O: C 75.78, H 3.89, N 14.73. Found; C 75.91, H 4.04, N 15.01. 

*Compound* **8**: mp 202–204 °C; ^1^H-NMR (DMSO-d_6_): *δ* 11.05 (1H,s), 7.93 (1H, d, *J *= 7.9), 7.44 (2H, s), 7.35 (1H, t, *J *= 7.3), 7.20–7.01 (4H, br m), 6.86 (1H, d, *J *= 6.9), 6.62 (1H, br d, *J *= 6.6), 6.57 (1H, t, *J *= 6.6), 6.40 (1H, br d, *J *= 6.4); ^13^C-NMR (DMSO-d_6_): *δ *103.3, 113.9, 114.9, 117.3, 118.5, 120.8, 122.7, 128.6, 129.3, 134.3, 135.6, 136.1, 138.1, 144.3, 152.7, 153,5, 189.2; IR (KBr): 3437, 3310, 1682 cm^−1^; MS: *m/z* 287 (M^+^). Anal. Calcd for C_18_H_13_ON_3_: C 75.24, H 4.56, N 14.63. Found: C 74.97, H 4.65, N 14.57. 

### 3.3. X-ray Crystal Structure Analysis

X-ray diffraction data were collected on a Rigaku RAXIS RAPID imaging plate area detector with graphite monochromated Mo-K*α* radiation (*λ *= 0.71075 Å). Diffraction data were collected at the temperature shown below. The crystal structures were solved by the direct method using SHELX97 for **2** and **8** and SIR92 for **3**, **5**, and **7** and refined by the full-matrix least-squares method. Non-hydrogen atoms were placed at calculated positions with C-H = 0.95 Å and refined using the riding model. All calculations were performed using the CrystalStructure 3.8.2 crystallographic software package [28,29]. Crystal data and other experimental details have been deposited at the Cambridge Crystallographic Data Centre (CCDC). **2**: CCDC 901409. **3**: CCDC 901410. **5**: CCDC 901411. **7**: CCDC 901412. 8: CCDC 901413. These data can be obtained free of charge via www.ccdc.cam.ac.uk/ conts/retrieving.html (or from the CCDC, 12 Union Road, Cambridge CB2 1EZ, UK; fax: +44 1223 336033; Email: deposit@ccdc.cam.ac.uk).

*Compound ***2**: C_19_H_12_O_2_N_2_, *M *= 300.32, triclinic, space group *P*-1 (#2), *a *= 5.2039(19), *b *= 10.457(5), *c *= 26.005(9) Å, *α *= 88.951(15), *β *= 88.755(11), *γ *= 80.940(15)°, *V *= 139.70(9), *Z *= 3, *D*_calc _= 1.071 gcm^−3^, *R* = 0.1504, *R_w_* = 0.4652. 23 °C.

*Compound ***3**: C_19_H_10_N_2_O, *M *= 282.30, monoclinic, space group *P*21/*n* (#14), *a *= 10.541(4), *b *= 8.865(4), *c *= 13.749(6) Å, *β *= 99.388(16)°, *V *= 1267.7(9) Å^3^, *Z *= 2, *D*calc = 0.739 gcm^−3^, *R* = 0.0434, *Rw* = 0.0557. −50 °C.

*Compound ***5**: C_62_H_43_N_9_O_2_, *M *= 946.08, triclinic, space group*P*-1 (#2), *a *= 12.409(4), *b *= 14.230(6), *c *= 15.949(7) Å, α = 116.175(13), *β *= 108.024(11), *γ *= 94.274 (13)°, *V *= 2328.9(16) Å^3^, *Z *= 2, *D*_calc _= 1.349 gcm^−3^, *R* = 0.1003, *Rw* = 0.1143. −80 °C. 

*Compound* **7**: C_18_H_11_N_3_O, *M *= 285.30, monoclinic, space group *P*21/*c* (#14), *a *= 5.0742(1), *b *= 16.0251(4), *c *= 15.7047(4) Å, *β *= 97.303(7)°. *V *= 1266.67(6) Å^3^. *Z *= 4, *D*calc = 1.496 gcm^−3^, *R* = 0.0385, *Rw* = 0.1073. −180 °C.

*Compound***8**: C_18_H_13_ON_3_, *M *= 287.32, triclinic, space group *P*-1 (#2), *a *= 8.043(4), *b *= 8.503(3), *c *= 10.632(4) Å, *α *= 85.011(18), *β *= 85.887(19), *γ *= 68.204(17)°, *V *= 672.0(5) Å^3^, *Z *= 2, *D*_calc_ = 1.420 gcm^−3^, *R* = 0.0370, *Rw* = 0.0981. −50 °C.

## 4. Conclusions

In summary, we report the formation of new spiro-*N,N*-ketal along with some unexpected rearranged compounds in the reaction of ninhydrin with naphthalene-1,8-diamine in the presence of an acid catalyst. The formation of such rearranged products is attributed to the activated electrophilicity of the C-1 carbon of ninhydrin in the presence of the acid catalyst. When isatin was reacted with naphthalene-1,8-damine along with a catalytic amount of acid, novel rearranged products were also isolated along with a spiro-*N,N*-ketal compound. The structures of the new spiro compounds were unambiguously determined on the basis of X-ray chrystalographic analysis results. 
